# Extracellular Non-Coding RNAs in Cardiovascular Diseases

**DOI:** 10.3390/pharmaceutics15010155

**Published:** 2023-01-03

**Authors:** Zeyidan Jiapaer, Chengyu Li, Xinyu Yang, Lingfei Sun, Emeli Chatterjee, Lingying Zhang, Ji Lei, Guoping Li

**Affiliations:** 1 College of Life Science & Technology, Xinjiang University, Urumqi 830046, China; 2 Xinjiang Key Laboratory of Biological Resources and Genetic Engineering, Urumqi 830046, China; 3 Fangshan Hospital Beijing University of Chinese Medicine, Beijing 102400, China; 4 Cardiovascular Research Center, Massachusetts General Hospital, Harvard Medical School, Boston, MA 02114, USA; 5 Center for Transplantation Science, Massachusetts General Hospital, Harvard Medical School, Boston, MA 02114, USA

**Keywords:** extracellular RNAs, non-coding RNAs, cardiovascular diseases, biomarkers, therapeutical targets

## Abstract

Cardiovascular diseases (CVDs) remain the world’s leading cause of death despite the best available healthcare and therapy. Emerging as a key mediator of intercellular and inter-organ communication in CVD pathogenesis, extracellular vesicles (EVs) are a heterogeneous group of membrane-enclosed nano-sized vesicles released by virtually all cells, of which their RNA cargo, especially non-coding RNAs (ncRNA), has been increasingly recognized as a promising diagnostic and therapeutic target. Recent evidence shows that ncRNAs, such as small ncRNAs, circular RNAs, and long ncRNAs, can be selectively sorted into EVs or other non-vesicular carriers and modulate various biological processes in recipient cells. In this review, we summarize recent advances in the literature regarding the origin, extracellular carrier, and functional mechanisms of extracellular ncRNAs with a focus on small ncRNAs, circular RNAs, and long ncRNAs. The pathophysiological roles of extracellular ncRNAs in various CVDs, including atherosclerosis, ischemic heart diseases, hypertension, cardiac hypertrophy, and heart failure, are extensively discussed. We also provide an update on recent developments and challenges in using extracellular ncRNAs as biomarkers or therapeutical targets in these CVDs.

## 1. Introduction

Cardiovascular diseases (CVDs) are widely recognized as the leading cause of death worldwide. Despite the considerable advances in both healthcare and therapies over the past decades, CVD has contributed to 17.9 million deaths in 2019 and is estimated to result in 22.2 million deaths in 2030 [1,2]. There is, therefore, an unmet medical need to develop novel diagnostics and therapeutics targeting CVDs. Emerging evidence suggests that extracellular non-coding RNAs (Ex-ncRNAs), particularly non-coding RNAs (ncRNAs) encapsulated in extracellular vesicles (EVs), function as key mediators of intercellular and inter-organ communication and play versatile roles in both homeostasis and disease [3]. EVs are continuously secreted by cells into circulation and are found in all biological fluids. The encapsulated molecular cargo inside EVs, especially RNAs, are increasingly recognized as promising biomarkers and therapeutic targets for various diseases, including CVDs [4,5,6].

**Figure 1:**
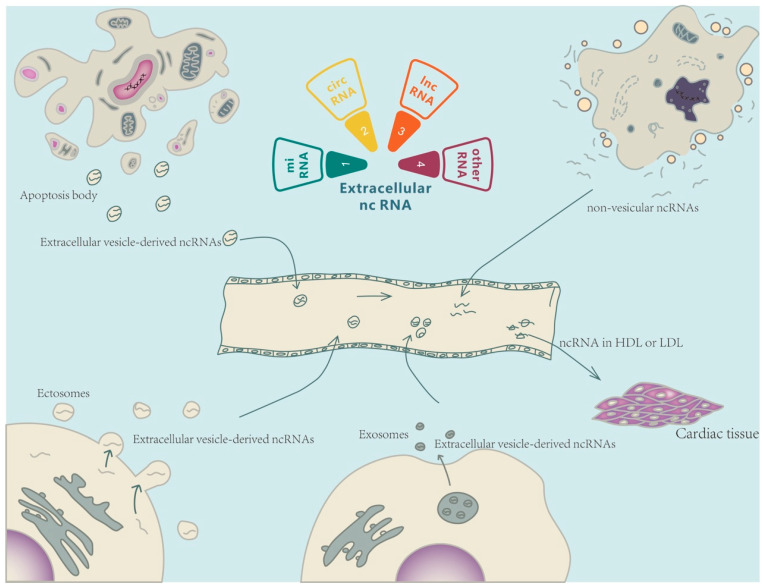
**Extracellular non-coding RNA-mediated intercellular crosstalk.** Extracellular non-coding RNAs (Ex-ncRNAs) are a heterogeneous group of RNAs and mainly include small ncRNAs, lncRNAs, and circRNAs. Ex-ncRNAs can be translocated into receipt cells and modulate the cellular functions of targeted cells (heart or other cardiovascular systems). Ex-ncRNAs can be secreted into the extracellular space through a variety of pathways, including embedded into exosomes, ectosomes, and apoptotic bodies, or partnered with ribonucleoproteins and lipoproteins.

Ex-ncRNAs are a heterogeneous group of RNAs, including small ncRNAs, long ncRNAs (lncRNAs), and circular RNAs (circRNAs), which encompass the majority of the extracellular transcriptome [7,8]. Ex-ncRNAs can be translocated into receipt cells after being secreted into the extracellular space through a variety of methods, thereby regulating various biological processes in targeted cells under both physiological and pathological conditions [9] (Figure 1). This review summarizes the recent updates of Ex-ncRNAs and discusses the diagnosis and therapeutic aspects of Ex-ncRNAs in CVDs, including atherosclerosis, ischemic heart diseases, hypertension, cardiac hypertrophy, and heart failure.

## 2. Ex-ncRNAs

### 2.1. Extracellular Small ncRNAs

The main classes of small ncRNAs are miRNAs, small interfering RNAs (siRNAs), piwi-interacting RNAs (piRNAs), Y-RNAs, and tRNA-derived small RNAs (tDRs) [10,11]. Most small ncRNAs are capable of regulating gene expression either transcriptionally or epitranscriptionally [10,12]. Recent evidence suggests that small ncRNAs are encapsulated into EVs and modulate the transcriptome of recipient cells following release through cellular autocrine or paracrine pathways. In addition, small ncRNAs, embedded in ribonucleoproteins and lipoproteins, are found to be secreted into extracellular spaces [13]. An increasing number of studies have detected extracellular small ncRNAs in a variety of biological fluids, such as serum or plasma, and indicated that extracellular small ncRNA profiles in various body fluids could serve as novel biomarkers for different pathological conditions [14,15,16,17]. As one of the most abundant RNA species in circulation, extracellular small ncRNAs are actively involved in a variety of pathological and physiological processes. Therefore, the detection of specific circulating small ncRNAs, based on the small ncRNA profiles, could be a promising approach to diagnosing diseases.

### 2.2. Extracellular LncRNAs

LncRNAs are a class of transcripts with a length of more than 200 nucleotides and lack protein-coding potential [18]. There are many categories and sub-categories of lncRNAs, with major classifications including antisense, bi-directional, enhancer-associated, intergenic lncRNAs (lincRNAs), and pseudogenes [19]. They function primarily in two fashions: (1) regulating the expression of miRNA target genes by mimicking miRNA sponges via competitive endogenous RNA (ceRNA) inhibition, and (2) regulating the post-translational modification of specific proteins, thereby affecting the activity of downstream signaling pathways [20]. Recent studies have noted that EVs are the primary means through which lncRNAs are transferred outside cells [21]. Extracellular lncRNAs can affect a broad range of downstream effects. In general, lncRNAs act as heterologous RNAs that can be transferred to target cells by EVs and regulate the cellular functions of receipt cells [22]. The membrane-bound nature of EVs shields ncRNA cargos from degradation. Due to the lack of evolutionary conservation and high specificity in different cells/organs, lncRNAs continue to be investigated as potential mediators of intercellular communication, exemplifying a valuable class of therapeutic targets for disorders as well as potential biomarkers [23].

### 2.3. Extracellular CircRNAs

CircRNAs are single-stranded RNAs that are linked end-to-end by a back-splicing mechanism. According to their splicing sequence, circRNAs can be categorized into the following groups: exonic circular RNAs (ecircRNAs), circular intronic RNAs (ciRNAs), exon-intron circular RNAs (EIciRNAs), intergenic circRNAs, anti-sense circRNAs, and tRNA intronic circRNAs (tricRNAs). Until now, the functionalities of circRNAs have been mainly classified into four categories: (1) acting as ceRNAs or sponges; (2) regulation of pre-RNA cleavage; (3) regulation of gene expression; and (4) as a potential source for translation of proteins and peptides [24]. Similar to the aforementioned ncRNAs, circRNAs can also be loaded into EVs to mediate cell-cell communication [13]. CircRNAs demonstrate a notable advantage over other ncRNAs. Without a linear terminal, circRNAs have a longer half-life, allowing for accumulation in tissues with a low proliferation rate. Additionally, the lack of a linear terminal impedes RNase degradation and improves stability and integrity in the extracellular environment, thus elevating their utility as biomarkers of disease [25]. Recent evidence has identified a crucial role of several extracellular circRNAs in alleviating damage due to cardiomyocyte hypertrophy, heart failure (HF), myocardial infarction (MI), and dysfunction caused by ischemia-reperfusion (I/R) [26]. Furthermore, several studies have also reported their association with proliferation, apoptosis, and inflammatory responses, thus influencing physiological and pathological phenomena in various tissues [7,27]. Investigation of circRNAs continues to elucidate their role in the pathogenesis of CVDs and provides a potential avenue for therapeutic development.

## 3. Ex-ncRNAs as Biomarkers in CVDs

In recent decades, several molecular mechanisms have been identified to be associated with the induction and progression of CVDs, especially in coronary atherosclerosis and HF [28]. Although clinical management of HF is improving, incidence rates remain above 20% in the adult population, with mortality rates hovering at 50% within 5 years of diagnosis, which makes HF a leading cause of morbidity and mortality in developed countries [29]. Consequently, it is necessary to delve into the disease’s underlying mechanisms and subsequent release of ncRNAs to develop more effective diagnostic and prognostic tools to reduce mortality (Table 1).

### 3.1. Atherosclerosis

Atherosclerosis is a chronic inflammatory artery disease characterized by the deposition of atherosclerotic plaque in the arteries, which leads to the hardening and narrowing of the artery lumen and subsequent obstruction of blood flow [30]. Atherosclerotic plaque results from the accumulation of circulating low-density lipoprotein (LDL) cholesterol alongside fibrous materials and inflammatory cells on the inner layers of arteries [31]. On the contrary, other cholesterol classes, namely high-density lipoproteins (HDLs), hold a significant anti-atherosclerotic role, transporting cholesterol from peripheral tissues to the liver for metabolism [32].

#### 3.1.1. Extracellular Small ncRNAs

In 2011, a study found that circulating HDLs and LDLs can transport miRNAs, such as HDL-transported miR-223, to regulate different intercellular signaling pathways in atherosclerosis [33,34]. In addition to cholesterol-driven miRNA transport, circulating miRNAs have also been identified as potential biomarkers of atherosclerosis in hypertensive patients. By comparing the expression levels of miR-92a in plasma of different levels of carotid intima-media thickness (CIMT) and hypertensive patients, a recent study found that the increased level of miR-92a expression was positively correlated with the measured value of CIMT, ambulatory blood pressure monitoring results and carotid-femoral pulse wave velocity, all symptoms of atherosclerosis [35]. Given that lipoproteins play an important role in the progression of atherosclerosis, differential expression of HDL-miRNAs, in addition to circulating miRNAs, can help track disease progression and enable more accurate diagnosis and treatment.

#### 3.1.2. Extracellular LncRNAs

Extracellular lncRNAs such as lncRNA Sox2 Overlapping Transcript (SOX2-OT) have also been shown to be potentially involved in the development of vascular disease [36]. Serum SOX2-OT levels were found to be significantly elevated in atherosclerotic patients. The results revealed that it could be used as a diagnostic biomarker and an excellent modality to assess patient prognosis [37].

#### 3.1.3. Extracellular CircRNAs

Another study quantified circRNA in the plasma of patients with coronary artery disease. It was reported that the plasma level of hsa_circ_0001445 was reduced in patients with coronary artery sclerosis, and the condition remained stable in patients with reduced expression of hsa_circ_0001445. The expression level of hsa_circ_0001445 was inversely correlated with the degree of coronary atherosclerosis. Combined with coronary computed tomography, its quantification significantly improved the diagnosis of the degree of disease [38].

### 3.2. Ischemic Heart Disease

Ischemic cardiomyopathy is caused by an imbalance in the myocardial oxygen demand and available oxygen supply, most often induced by coronary artery stenosis. Based on the pathophysiology, ischemic cardiomyopathy includes coronary heart disease and myocardial infarction (MI) [39].

Although stenting is a crucial method to relieve myocardial ischemia, it may result in long-term cardiac damage when blood flow is suddenly restored, also known as ischemia-reperfusion (I/R) injury [40,41]. I/R injury after reperfusion often occurs due to cardiomyocytes undergoing anaerobic metabolism, sodium-potassium pump dysfunction, and ribosome shedding. Various intracellular ATP-dependent ion pumps become inactive, leading to ion accumulation, pH down-regulation, and up-regulation of intracellular osmolarity. Ultimately, ischemic cardiomyocytes experience cell swelling, impaired enzyme activities, and clumped nuclear chromatin. Due to the low concentration of antioxidants in ischemic cardiomyocytes, the increased reactive oxygen species after reperfusion causes oxidative stress, which further causes cellular dysfunction, DNA damage, and local inflammatory responses in cardiomyocytes. Thus, the persistence of inflammation and oxidative stress may trigger a cytokine storm that exposes cells to severe damage [42].

#### 3.2.1. Extracellular Small ncRNAs

Currently, the primary diagnosis of MI is mainly based on the detection of MI markers like cardiac troponin [43,44]. However, there is evidence showing that elevated cardiac troponins are present outside cardiac injury and can compromise the diagnosis of MI [45]. This opens an avenue to pursue Ex-ncRNA levels as a circulating biomarker for acute myocardial infarction (AMI) [46]. A previous study observed a 1600-fold elevated expression of miR-208b in AMI compared to the control group. Further, miR-208b identified earlier detection than troponin T regarding MI progression. In addition, another study found that miR-204 was down-regulated, and lncRNA-NEAT1 and matrix metalloproteinase-9 (MMP-9) were upregulated in serum EVs from patients with acute ST-segment elevation MI [47]. Serum-derived miRNA signatures have been used to distinguish healthy volunteers from patients with coronary atherosclerosis through the expression levels of miR-370-3p and miR-409-3p, which can serve as a fingerprint for coronary heart disease [48]. By exploring the expression levels of EV-derived miRNAs in the plasma and serum of CS and AMI patients, EV miRNA landscapes facilitated disease differentiation [49].

Novel miRNAs have been found to identify the evolution of cardiac I/R injury [50]. MiRNAs with diagnostic or therapeutic potential have been identified in the context of early cardiac I/R injury with miRNA arrays conducted to screen for differential expression in a mouse model of cardiac I/R injury. A total of 1882 miRNAs were screened, among which 11 were observably down-regulated and 41 were markedly up-regulated 3 h after reperfusion. miR-3113-5P and miR-223-3p were among the most differentially expressed miRNAs and have been confirmed to be up-regulated in the early stage of cardiac tissue I/R injury [51,52]. This data confirms that cardiac miRNAs, such as miRNA-3113-5p, might be a valuable target for therapeutic purposes, and circulating miRNAs such as miRNA-3113-5p might serve as a stable marker for the early diagnosis of cardiac I/R injury.

Extracellular tDR is a newly identified small regulatory RNA species. A recent study has demonstrated that extracellular tDRs are much more dynamically regulated than intracellular tDRs and extracellular miRNAs in both cardiomyocytes and cardiac fibroblasts upon the treatments of ischemia/reperfusion-related stressors [11]. Notably, more than 3000 extracellular tDRs are significantly regulated by nutritional deprivation or ischemia, and approximately 2000 extracellular tDRs are differentially expressed upon ischemia/reoxygenation treatment mimicking ischemia/reperfusion injury in both cardiomyocytes and cardiac fibroblasts [11]. Detailedly, extracellular tDR-1:32-His-GTG-1, tDR-37:72-Val-TAC-1, tDR-1:32-Pro-AGG-1-M4, tDR-2:30-Glu-CTC-1, tDR-2:30-Glu-CTC-1-D4G, tDR-1:31-Glu-TTC-4, tDR-3:31-Gly-GCC-2-M2, and tDR-40:72-Asn-GTT-1-M2 were significantly induced by ischemia/reoxygenation from both cardiomyocytes and cardiac fibroblasts, and extracellular tDR-1:36-Glu-CTC-1, tDR-1:36-Glu-CTC-1-D5G, tDR-1:36-Asp-GTC-2-M2, and tDR-42:75-Ser-GCT-3 were downregulated considerably upon cardiac ischemia/reoxygenation [11]. Although this study is a preliminary cell culture model, the findings clearly suggest that the extracellular tDR may be a promising biomarker for diagnostic and prognostic assessments of ischemic heart diseases.

#### 3.2.2. Extracellular LncRNAs

A previous study has shown that lncRNAs can also serve as potential biomarkers for AMI [53]. It was found that the level of circulating lncRNA ENST000005566899.1 and lncRNA ENST00000575985.1 were significantly elevated in the plasma-derived EVs of AMI patients compared to healthy individuals. Further study of these lncRNAs in combination with mature biomarkers will enhance the understanding of disease progression which is fundamental for developing lncRNA therapeutics treating MI. Owing to the similarities of disease presentation and the vast differences in management, it becomes imperative to pursue avenues that allow for identifying the types and stages of diseases using noninvasive biomarkers.

#### 3.2.3. Extracellular CircRNAs

Previous studies have shown that the downregulation of circRNA MICRA in peripheral blood is associated with the risk of left ventricular remodeling and dysfunction after MI. Additionally, the upregulation of hsa-circ-0098964 and circRNA-284 in the serum has been found to be associated with an increased risk of hypertension and acute ischemia [54].

### 3.3. Hypertension

Hypertension is a significant cause of heart disease and death worldwide [55]. As the heart pumps blood, the blood applies outward pressure on the arterial wall. The maximum pressure exerted while beating is defined as systolic pressure. Conversely, blood pressure on the arterial wall during relaxation and dilatation of the heart muscles is known as diastolic pressure. Hypertension is diagnosed when systolic pressure exceeds 140 mmHg, or diastolic pressure exceeds 90 mmHg [56]. One of the leading causes of hypertension is vascular dysfunction which manifests as endothelial dysfunction, vascular inflammation, arterial remodeling, and lowered vascular elasticity [57].

#### Extracellular Small ncRNAs

Extracellular miR-133a, miR-21, and miR-27a have previously been reported to play an essential role in the development of hypertension [58,59,60]. Based on these findings, a study was conducted on a general population over five years, measuring the serum levels of miR-133a, miR-21, and miR-27a using RT-qPCR. It was found that the serum expression levels of miR-133a and miR-27a were negatively correlated with the incidence of hypertension and, as a result, could be used as diagnostic biomarkers and preventive and predictive biomarkers of hypertension [61]. Recently, a study using serum from hypertensive patients quantified miR-92a and found that its expression was significantly increased in the serum of hypertensive patients, verifying its potential as a biomarker [62]. An in vitro study further supports the potential of extracellular miR-92a as a biomarker of hypertension as it targets vascular smooth muscle cells to regulate vascular SMC phenotype, thereby causing arterial stiffness and participating in the occurrence and development of hypertension [62].

Hypertensive disorder during pregnancy is a common type of hypertension [63,64]. Commonly, severe pregnancy-induced hypertension causes irreversible damage to the mother and offspring. Previous studies have revealed that miR-200a-3p can coordinate the function of trophoblast [65]. Based on this conclusion, one study quantified miR-200a-3p in the serum of hypertensive patients during pregnancy by RT-qPCR and found that the expression level of miR-200a-3p in the serum of hypertensive patients during pregnancy was up-regulated. Moreover, its expression level was positively correlated with the development of the disease. As a result, the level of miR-200a-3p bears prognostic potential for gestational hypertension [66].

### 3.4. Heart Failure

HF is a clinical syndrome of structural or functional abnormalities of the heart [67]. As HF may not have symptoms at its early stage, early screening using biomarkers can significantly reduce the risk of HF. The current standard for diagnosis utilizes the N-terminal-proB-type Natriuretic Peptide (NT-proBNP) test. However, increased concentrations of BNPs are not always associated with the onset of HF and may impede accurate and earlier diagnosis of HF.

#### 3.4.1. Extracellular Small ncRNAs

A previous study has examined the expression of Ex-ncRNAs in plasma in relation to the progression of HF [68], and found altered expression of miR-192, miR-194, and miR-134a in the plasma from patients with HF, implying the capacity of small ncRNAs to identify cardiomyocyte death and HF [68]. Similarly, another study showed that paracrine signaling via cardiomyocyte-derived EVs containing miR-30d could improve cardiac function by reducing myocardial fibrosis and cardiomyocyte apoptosis [69]. Small ncRNAs have also been assessed in the serum of acute HF patients following dilated cardiomyopathy. In this cohort, miR-92b-5p expression was up-regulated in the EVs of the patients, suggesting its use as a potential biomarker in diagnosing acute AHF caused by dilated cardiomyopathy [70].

#### 3.4.2. Extracellular LncRNAs

Ischemic cardiomyopathy (ICM) caused by MI is a major cause of HF. In the context of circulating biomarkers, lncRNAs in ICM have been widely explored. Genome-wide transcriptome analysis has verified that a number of protein-coding genes previously reported to be associated with HF demonstrated altered expression following ICM [71,72]. Among 145 differentially expressed lncRNAs screened in ICM, 35 lncRNAs showed strong positive correlations. Expression correlation coefficient analysis of differentially expressed lncRNAs and the protein-coding genes yielded a strong correlation between lncRNAs and ECM protein-coding genes. The overexpression or knockdown of selected lncRNAs in the cardiac fibroblasts indicated that lncRNAs were significant regulators of fibrosis and ECM synthesis gene expression [73,74]. In addition, lncRNAs were found to be involved in the TGF-β pathway to regulate ECM gene expression and myofibroblast differentiation [75]. This data suggests that lncRNAs might be novel modulators of heart function and HF.

**Table 1:** Ex-ncRNAs as biomarkers in CVDs.

Reference	Ex-ncRNA	Carriers	Expression (↑) (↓)		Type of CVDs
Kasey et al. [33]	miR-223	HDL	↑	-	Atherosclerosis
Huang et al. [35]	miR-92a	-	↑	-	Atherosclerosis
Tao et al. [37]	LncRNA SOX2-OT	-	↑	-	Atherosclerosis
Vilades et al. [38]	hsa_circ_0001445	-	-	↓	Atherosclerosis
Corsten et al. [46]	miR-208b	-	↑	-	Acute myocardial infarction
Li et al. [11]	tDR-1:32-His-GTG-1, tDR-37:72-Val-TAC-1, tDR-1:32-Pro-AGG-1-M4, etc.	-	↑		Cardiac ischemia/reperfusion
	tDR-1:36-Glu-CTC-1, tDR-1:36-Glu-CTC-1-D5G, tDR-1:36-Asp-GTC-2-M2, etc.	-		↓	
Chen et al. [47]	LncRNA-NEAT1, miR-204	EV	↑	↓	Acute ST-segment elevation myocardial infarction
Hildebrandt et al. [48]	miR-370-3p, miR-409-3p	EV	↑	-	Coronary heart disease
Chen et al. [50]	microRNA 3113-5P	-	↑	-	Cardiac ischemia/Reperfusion injury
Zheng et al. [53]	lncRNA ENST00000556899.1, lncRNA ENST00000575985.1	EV	↑	-	Acute myocardial infarction
Kishore et al. [54]	circRNA MICRA, circRNA-284, hsa-circ-0098964	-	↑	↓	Myocardial infarction, Ischemic heart disease, Hypertension
Suzuki et al. [61]	miR-133a, miR-27a	-	-	↓	Hypertension
Wang et al. [62]	miR-92a	-	↑	-	Hypertension
He et al. [66]	miR-200a-3p	-	↑	-	Hypertension
Janjusevic et al. [68]	lncRNA LIPCAR, miR-192, miR-134a, miR-194, miR-30d	EV	↑	↓	Heart failure
Li et al. [69]	miR-30d	EV	-	↓	Heart failure
Wu et al. [70]	miR-92b-5p	EV	↑	-	Heart failure

## 4. Ex-ncRNAs as Therapeutic Targets in CVDs

Over the past decade, substantial effort has been made toward better understanding the functional roles of Ex-ncRNAs in CVDs and the clinical applications of novel therapies targeting Ex-ncRNAs, based on the approaches modulating their expression or their interactions with targets. The therapeutic functions of most of the small ncRNAs can be induced by synthesized RNA oligos, known as mimics, with multiple chemical modifications that improve pharmacokinetics and pharmacodynamics [76]. With the recent success of two COVID-19 mRNA vaccines [77], long RNA mimics have been emerging as promising therapeutics to induce functional effects of lncRNAs in patients. Besides the synthetic circRNA mimics, circRNAs are usually overexpressed by gene-expressing viral or non-viral vectors [78], which can also be used to express some small and long ncRNAs. The tools exploiting the exquisite sensitivity of base-pair complementarity have been used extensively to inhibit the functions of disease-causing Ex-ncRNAs [76,78,79], which include antisense oligonucleotides (ASOs), small interfering RNAs (siRNAs), short hairpin RNAs (shRNAs), antisense, miRNA sponges, CRISPR/Cas9-based DNA editing tools, and CRISPR/Cas13-based RNA editing tools. Here, we describe recent studies that targeted Ex-ncRNAs in various CVDs (Table 2).

### 4.1. Atherosclerosis

#### 4.1.1. Extracellular Small ncRNA

It is well-known that inflammation plays a vital role in the development of coronary atherosclerotic diseases (CAD) [80,81]. Evidence suggests that intercellular crosstalk of Ex-ncRNAs can influence the development of inflammation and subsequent CAD progression. EV-encapsulated miRNA-146a-5p treatment resulted in production of proinflammatory cytokines, injury of the coronary endothelial cell barrier, and cardiomyocyte dysfunction, by targeting inflammation-triggering Toll-like receptor 7 (TLR7) [82]. Other avenues facilitating atherosclerosis progression include Ex-ncRNAs acting as danger-associated molecular patterns (DAMPs) through stimulation of the vascular endothelial growth factor (VEGF) receptor-2 system to increase vascular permeability and inflammatory cell aggregation [83].

#### 4.1.2. Extracellular LncRNAs

A previous study has shown that lncRNA-RNCR3 is significantly upregulated during atherosclerosis. RNCR3 acts as a ceRNA for miR-185-5p, leading to the upregulation of its target gene KLF2, a critical transcription factor for endothelial vasoprotection. Targeting RNCR3 may thereby protect from atherosclerosis-related injury [84].

#### 4.1.3. Extracellular CircRNAs

The studies focusing on circRNAs for therapeutic application have identified a wide range of potential targets against atherosclerosis development and progression. A recent study reported the specific mechanism of hsa_circ_0001445 on endothelial injury following treatment with oxidized LDL (ox-LDL) [85]. hsa_circ_0001445 was found to directly target miR-208b-5p and lead to the upregulation of ABCG1, the direct target of miR-208b-5p [85]. The study concludes that targeting hsa_circ_0001445 promotes aortic endothelial cell proliferation and migration through the mir-208b-5p/ABCG1 axis and inhibits inflammation and extracellular matrix (ECM) to reduce the damage of ox-LDL to endothelial cells (ECs) [85].

As most Ex-ncRNAs, except circRNAs, contain linear terminals, targeting ncRNAs using RNases presents a route for therapeutic inhibition. RNases are capable of hydrolyzing Ex-ncRNAs. In linear ncRNAs, such as small and lncNRAs, RNase digestion can inhibit the development of arterial inflammation and suppress atherosclerosis [86]. Investigations on RNase-based therapies targeting Ex-ncRNAs have begun to reveal yet another critical avenue of ncRNA therapeutics [87].

### 4.2. Ischemic Heart Disease

#### 4.2.1. Extracellular Small ncRNAs

In the immune microenvironment, EVs produced by immune cells can selectively carry Ex-ncRNAs that affect the development of MI [88]. Among the highly expressed pro-inflammatory miRNAs, miR-155 was found to inhibit angiogenesis and promote cardiac dysfunction by downregulating Rac family small GTPase 1 (RAC1), p21-activated kinase 2 (PAK2), Sirtuin 1 (Sirt1), and protein kinase AMP-activated catalytic subunit alpha 2 (AMPKα2) [89]. These proteins subsequently reduce the ability of ECs to induce angiogenesis and advance inflammation, leading to the exacerbation of MI [89]. In another vein, miR-1271-5p, carried by M2-like macrophage-derived EVs, plays a protective role in AMI [90]. It directly affects the expression of SRY-Box transcription factor 6, which prevents cardiomyocyte damage and promotes cardiac repair and cardiomyocyte viability [90]. Additionally, miR-30d, the HF biomarker mentioned above, may potentially target integrin 5 and mitogen-activated protein kinase 4 (MAPK4) in cardiac fibroblasts in response to acute cardiac damage [69].

In addition to macrophages, dendritic cells (DCs) also play an essential role in the immune microenvironment after MI. This study has found that miR-494-3p could be loaded into DC-derived EVs and promote post-MI angiogenesis by inducing VEGF expression in mice [91]. Mesenchymal stem cells (MSCs) derived Ex-ncRNAs also have been found to play a crucial role in intercellular communication. Overexpression of miR-486-5p, derived from MSCs, silences MMP19 and promotes the activity of ECs to induce angiogenesis [92]. As such, MSC EVs may be potentially used to promote angiogenesis by delivering miR-486-5p-loaded EVs. This modality may be a critical therapeutic approach in myocardial ischemic compensation [92]. MSC-derived EV transfer of miR-210 has also been found to protect cardiomyocytes from apoptotic cell death, with miR-210 downregulating the expression level of apoptosis-inducing factor mitochondria associated 3 (AIFM3) and affecting PI3K/AKT and p53 signaling pathways to reduce apoptosis in cardiomyocytes and improve cardiac function [93]. To a certain extent, MSCs under hypoxic conditions offer significant protection against I/R injury. Upregulated miR-224-5p in mouse adipose, MSC EVs were found to target thioredoxin-interacting protein (TXNIP), thereby inhibiting the degradation of GATA binding protein 4 (GATA4) and maintaining the expression of Bcl-2 [94]. TXNIP was also identified as a target for miR-150-5p [95]. Therefore, as expected, overexpression of miR-150-5p protected rat hearts during I/R by inhibiting the expression of TXNIP.

The persistence of inflammation and oxidative stress may trigger a cytokine storm that exposes cells to severe damage [42]. Bone marrow mesenchymal stem cells (BMSC) EVs exert protective effects at different levels of oxidation [96]. It is worth noting that the miR-29c was upregulated in BMCS EVs and protected against hypoxia/reoxygenation (H/R) injury by inhibiting the PTEN/Akt/mTOR axis. Additionally, two extracellular miRNAs: miR-149 and let-7c, were significantly upregulated in EVs from normal BMSCs compared to H/R BMSCs [97]. These two upregulated miRNAs downregulate the expression of the Faslg gene by promoting the miR-149/Let-7c/FASLG axis [97]. Another study using BMSC-derived EVs has shown that miR-338 is highly enriched in BMSC EVs [98]. Upregulation of miR-338 inhibits MAP3K2 gene expression by suppressing the MAP3K2/JNK axis, thus inhibiting apoptosis in cardiomyocytes. As such, it can be concluded that BMSC EVs have the potential to ameliorate cardiac conditions during MI.

Cardiac cell-derived EVs encapsulate many miRNAs like miR-146a, miR-181b, and miR-26a, and have exhibited improved cardio-protective and therapeutic effects than MSC EVs under MI conditions [99,100,101,102]. Using single RNA tracing, cardiac endothelial cells and cardiac fibroblasts have shown increased intake of cardiosphere cell-derived EVs following injury [103]. Several cardiomyocyte EC-derived miRNAs such as miR-23a-3p, miR-424, let-7f, miR-378, and miR-214 might be one of the crucial cardio protecting factors [104]. Moreover, evidence suggests that miR-19a-3p can downregulate hypoxia-inducing factor-1α (HIF-1α), leading to the inhibition of cardiomyocyte proliferation and angiogenesis. Administration of the miR-19a-3p antagomir has downregulated miR-19a-3p expression, accelerated angiogenesis, and exerted a protective effect after MI [105]. Another study has shown that the downregulated expression of miR-143 in cardiomyocyte-derived EVs leads to the induction of angiogenesis and reduces cardiac ischemic pressure through the IGF-IR/NO signaling pathway [106]. Through a small RNA sequencing study, cardiac telomeric cell-derived EVs have demonstrated the transport of miR-21-5p to ECs, where it silences Cdip1 gene expression. Silencing of Cdip1 downregulates caspase-3 protein expression and promotes angiogenesis, subsequently suppressing ischemic cardiomyopathy [107].

#### 4.2.2. Extracellular LncRNAs

It is reported that human BMSC EVs containing lncRNA HCP5 could protect cardiomyocytes from I/R injury by inhibiting the IGF1/PI3K/AKT axis [108]. In detail, it functions as a sponge for endogenous miR-497, which targets the expression of IGF-1 [108]. Another study using human umbilical cord mesenchymal stem cell (ucMSC)-derived EVs to treat H/R cardiac microvascular ECs and I/R rats has found that human ucMSC-derived EVs containing the lncRNA UCA1 reduces the injury of cardiac microvascular ECs in vivo [109]. UCA1 sponges miR-143 and subsequently inhibits Bcl-2 via the miR-143/Bcl-2/Beclin-1 axis [109].

#### 4.2.3. Extracellular CircRNAs

Researchers have found that circRNAs also have protective effects against MI. For example, circ-HIPK3-rich EVs were released from cardiomyocytes under hypoxic conditions and protected the heart from oxidative stress injury [110]. Circ-HIPK3 sponges miR-29a and promotes the expression of vascular endothelial growth factor A (VEGFA), accelerating the proliferation, migration, and angiogenesis of ECs. Upregulation of VEGFA thereby reduces the effects of MI and protects against MI [110]. Another study used microarray analysis to establish the circRNA expression profile of EVs derived from M2-like macrophages. This was followed by the identification, and functional characterization of M2-like macrophage-derived EVs in MI mice models [111]. From this, it was found that highly expressed circRNA-UB3A can enter cardiac fibroblasts to sponge the expression of miR-138-5p. This results in the inhibition of Rho C expression, which exacerbates myocardial fibrosis after MI [111]. In summary, Ex-ncRNAs (miRNAs, lncRNAs, and circRNAs) derived from various resources (cardiomyocytes, stem cells, immune cells) mediate crosstalk between cell-to-cell through a variety of modalities, affecting the proliferation and apoptosis of cardiomyocytes alongside promoting angiogenesis. At the same time, they can counteract the generation of ischemic injury of cardiomyocytes.

### 4.3. Hypertension

#### Extracellular Small ncRNAs

Endothelial dysfunction is a significant manifestation of hypertension. It was shown that miR-483 could target transforming growth factor-β (TGF-β), TGF-β receptor 2 (TGFBR2), β-catenin, connective tissue growth factor (CTGF), interleukin-1β (IL-1β), and endothelin-1 (ET-1) to obtain anti-hypertensive effects [112]. The protective mechanisms mediated by extracellular miR-483-3p derived from ECs in hypertension were also elucidated [113]. This study showed that miR-483-3p was associated with the progression of hypertension and that the expression level of miR-483-3p in serum was proportional to vascular function. Overexpression of miR-483-3p reduced the expression of TGF-β, CTGF, angiotensin-converting enzyme 1 (ACE1), and ET-1 genes both in ECs and smooth muscle cells (SMCs). Therefore, extracellular miR-483-3p may become one of the promising therapeutic targets for the treatment of various types of endothelial dysfunction represented by hypertension [113].

MiR-634, as a circulating biomarker, has been implicated in playing a role in pulmonary hypertension as well as systemic hypertension [114,115]. It was found that the expression level of miR-634 was reduced in the plasma of hypertensive patients, and miR-634 could target HASMCs through the Wnt/β-catenin signaling pathway and downregulate the expression of Wnt4, thereby inhibiting the proliferation and migration of HASMCs and influencing the course of hypertension by affecting vascular remodeling [116].

### 4.4. Cardiac Hypertrophy

Cardiac hypertrophy is mainly caused by changes in the mechanical stress of the heart. It is a compensatory mechanism of the heart to resist the increased hemodynamic pressure. The process of myocardial hypertrophy is primarily characterized by the enlargement of cardiomyocytes along with myocardial fibrosis and inflammation, resulting in myocardial dysfunction [117,118].

#### Extracellular Small ncRNAs

The process of myocardial hypertrophy is primarily characterized by the enlargement of cardiomyocytes along with myocardial fibrosis and inflammation, resulting in myocardial dysfunction [117,118]. It has been reported that miR-217 expression was increased in the hearts of thoracic aortic constriction (TAC) mice and CHF patients. In cases of stress overload and miR-217 overexpression in cardiac hypertrophy, fibrosis, and dysfunction, effects were reversed via miR-217-TUD-mediated miR-217 downregulation. It has been established that miR-217 directly targets PTEN, a protein involved in cell cycle regulation and proliferation. Importantly, exosomes produced from cardiomyocytes containing miR-217 promoted fibroblast growth in vitro. Following its implications in cardiac fibrosis and hypertrophy through control of PTEN, miR-217 is undoubtedly highlighted as a potential therapeutic target for chronic HF [119].

MiR-21-3p has been identified as upregulated in cardiac fibroblast-derived EVs by miRNA profiling assay and qRT-PCR. Exploration of the crosstalk between cardiac fibroblasts and cardiomyocytes in a co-culture system further validated the paracrine function of cardiac fibroblast-derived miR-21-3p on cardiomyocytes. Co-incubation of fibroblast-derived miR-21-3p and cardiomyocytes further confirmed the capacity of miR-21-3p to mediate cardiomyocyte hypertrophy. This study further demonstrates that miR-21-3p produced by cardiac fibroblasts silenced the expression of SH3 domain-containing protein 2 (SH3P2) and the PDZ and LIM domains in cardiomyocytes, encouraging hypertrophy and fibrosis. As a result, miR-21-3p may be a crucial therapeutic target for preventing cardiac hypertrophy [120]. On the other hand, miR-21-5p is known to regulate the ERK-MAP kinase signaling pathway in cardiac fibroblasts and influence the cardiac structure and function [121,122,123]. Interestingly, miR-21-5p levels are selectively increased in the fibroblasts of failing hearts and have enhanced ERK-MAP kinase activity by inhibiting sprouting homolog 1 (Spry1) [121]. In a murine model of pressure overload-induced cardiac hypertrophy, silencing of miR-21-5p by miRNA inhibitors suppressed the cardiac ERK-MAP kinase activity, attenuated interstitial fibrosis, and alleviated cardiac dysfunction [121]. Therefore, miR-21 served as the disease target for myocardial fibrosis and cardiac hypertrophy and defined the therapeutic efficacy of microRNA therapeutic interventions in CVDs.

The inflammatory immune microenvironment plays an important role in the development of myocardial hypertrophy. In parallel to the micro-immune microenvironment, immune-derived exosomes have become effective regulators of inflammatory response [124]. A study confirmed that exosomes derived from angiotensin II (Ang II)-induced hypertrophic cardiomyocytes (HCs) disturbed inflammatory signaling pathways in the macrophages [125]. The incubation of mouse macrophage cell line RAW264.7 in the presence of exosomes derived from HC medium activated the secretion of inflammatory cytokines interleukin (IL)-6 and IL-8 when compared with the exosomes derived from normal cardiomyocytes (NCs). The cytokine release triggered by exosomes derived from HCs was prevented by Argonaute2 (AGO2) down-regulation, suggesting that ncRNAs were involved in the exosome-induced inflammation in the RAW 264.7 macrophages. RNA sequencing further revealed that a total of seven microRNAs were differentially expressed between NCs-derived and HCs-derived exosomes, of which miR-155 plays a crucial role in the initiation of macrophage inflammation [126]. Further analysis showed that the HC-derived exosomes abducted the phosphorylation of ERK, c-Jun N-terminal kinase (JNK), and p38 through miR-155 [126]. These findings support that exosomal microRNA has become an important inflammatory response regulator in adjusting cardiac hypertrophy.

Cardiac hypertrophy, specifically myocardial hypertrophy, is often exacerbated by valvular calcification, a progressive disease prevalent in elderly individuals. Early intervention in the development of valvular calcification is a crucial measure to prevent myocardial hypertrophy [127,128]. In the cardiac interstitium, telocytes establish a complex association between cardiac stem cells and cardiomyocytes. When valvular calcification occurs, telocytes-derived EVs modulate myocardial regeneration, reduce myocardial fibrosis, and restore some functions [129]. Extraction of T telocytes EVs and knockdown of miR-30b expression revealed that inhibition of miR-30b could reduce calcium deposition in the valve, protecting the aortic valve and mitigating cardiac hypertrophy [129].

### 4.5. Heart Failure

#### 4.5.1. Extracellular Small ncRNAs

HF is a complex and progressive disease that may be caused by a variety of pathological conditions. Previous studies have indicated that Ex-ncRNAs also participate in the progression of HF. For example, a recent study demonstrated that the expression level of miR-21-5p was dysregulated in cardiac stromal cell-derived EVs from HF patients using microRNA arrays and qPCR analysis [130]. When the miR-21-5p level is downregulated, the repair effect in HF patients diminishes as it inhibits phosphatase and Tensin homologs to enhance AKT activity, thus causing the promotion of angiogenesis and protection of cardiomyocytes. This offers a promising therapeutical target for treating HF.

#### 4.5.2. Extracellular LncRNAs

LncRNAs are significant in heart development and disease. One study verified exercise-regulated cardiac lncRNA, called lncExACT, that was evolutionarily conserved and reduced in the exercising heart but augmented in the human and experimental HF [131]. The cardiac lncExACT1 overexpression led to pathological hypertrophy and HF. LncExACT1 suppressed physiological hypertrophy and cardiomyogenesis, preventing cardiac fibrosis and dysfunction. Further, lncExACT1 regulated microRNA-222, calcineurin signaling, and Hippo/Yap1 signaling vis DCHS2 [132,133]. Overexpression of DCHS2 in the zebrafish cardiomyocytes led to pathological hypertrophy and impaired cardiac regeneration, thereby promoting scar formation after injury [134]. On the contrary, DCHS2 depletion caused physiological hypertrophy and accelerated cardiomyogenesis in mice. These data demonstrated that lncExACT1-DCHS2 was identified as a new pathway for regulating HF.

**Table 2:** Ex-ncRNAs as therapeutic targets in CVDs.

Reference	Donor Cells	Ex-ncRNAs	Carriers	Expression Quantity (↑) (↓)		Target/Pathway	Type of CVDs
Shimada et al. [82]	-	miR-146a-5p	EV	↑	-	TLR7	Atherosclerosis
Shan et al. [84]	Human umbilical vein endothelial cells	lncRNA-RNCR3	EV	↑	-	miR-185-5p/KLF2	Atherosclerosis
Yang et al. [85]	-	hsa_circ_0001445	-	-	↓	mir-208b-5p/ABCG1	Atherosclerosis
Liu et al. [89]	M1-like macrophage	miR-155	EV	↑	-	Sirt1/AMPKα2 and RAC1-PAK2	Acute myocardial infarction
Long et al. [90]	M2-like macrophage	miR-1271-5p	EV	↑	-	SOX6	Acute myocardial infarction
Li et al. [69]	Cardiomyocytes	miR-30d	EV	↑	-	MAP4K4 and integrin α5	Ischemic HF
Liu et al. [91]	Dendritic cell	miR-494-3p	EV	↑	-	VEGF	Myocardial infarction
Li et al. [92]	MSC	miR-486-5p	EV	↑	-	MMP 19/VEGF	Myocardial infarction
Cheng et al. [93]	MSC	miR-210	EV	↑	-	AIFM3/PI3K/AKT and p53	Myocardial infarction
Mao et al. [94]	MSC	miR-224-5p	EV	↑	-	TXNIP/GATA4/Bcl-2	Ischemia-reperfusion injury
He et al. [95]	MSC	miR-150-5p	EV	↑	-	TXNIP	Ischemia-reperfusion injury
Li et al. [96]	BMSC	miR-29c	EV	↑	-	PTEN/Akt/mTOR	Ischemia-reperfusion injury
Zou et al. [97]	-	miR-149, let-7c	EV	↑	-	miR-149/Let-7c/FASLG	Ischemia-reperfusion injury
Fu et al. [98]	BMSC	miR-338	EV	↑	-	MAP3K2/JNK axis	Myocardial infarction
Walravens et al. [99,100,101,102]	CDC	miR-26a, miR-146a, miR-181b	EV	↑	-	Adam17, TLR-NFkB, PKCδ	Myocardial infarction
Moghiman et al. [104]	MSC, Cardiomyocyte, EC	miR-23a-3p, miR-424, let-7f, miR-378, miR-214	EV	↑	-	HOXA5, GAX, p38 MAPK, Smad2/3, ATM	Myocardial infarction
Gou et al. [105]	Cardiomyocyte	miR-19a-3p	EV	↑	-	HIF-1α	Myocardial infarction
Geng et al. [106]	Cardiomyocyte	miR-143	EV	-	↓	IGF-IR/NO	Myocardial infarction
Liao et al. [107]	CT	miR-21-5p	EV	↑	-	Cdip1/caspase-3	Myocardial infarction
Li et al. [108]	BMSC	lncRNA HCP5	EV	↑	-	miR-497/IGF1/PI3K/AKT	Ischemia-reperfusion injury
Diao et al. [109]	hUCMSC	lncRNA UCA1	EV	↑	-	miR-143/Bcl-2/Beclin-1	Ischemia-reperfusion injury
Wang et al. [110]	Cardiomyocyte	circ-HIPK3	EV	↑	-	miR-29a/VEGFA	Myocardial infarction
Wang et al. [111]	M2-like macrophage	circRNA-UB3A	EV	↑	-	miR-138-5p/RhoC	Myocardial fibrosis after myocardial infarction
Zhang et al. [112]	-	miR-483	-	-	↓	TGF-β, TGFBR2, β-catenin, CTGF, IL-1β, ET-1	Hypertension
Shang et al. [113]	-	miR-483-3p	EV	↑	-	TGF-β, CTGF, ACE1, ET-1	Hypertension
Niu et al. [116]	-	miR-634	-	-	↓	Wnt/β-catenin	Hypertension
Xiang et al. [119]	Cardiomyocyte	miR-217	EV	↑	-	PTEN	Cardiac hypertrophy
Claudia et al. [120]	Cardiac fibroblast	miR-21-3p	EV	↑	-	SH3 domain-containing protein 2 and PDZ and LIM domain 5	Cardiac hypertrophy
Thum et al. [121]	-	miR-21	-	↑	-	ERK-MAPK and Spry1	Cardiac hypertrophy
Yu et al. [125]	Macrophage	miR-155	EV	↑	-	Son of Sevenless 1 and Suppressor of Cytokine Signaling 1	Cardiac hypertrophy
Yang et al. [129]	Telocyte	miR-30b	EV	↑	-	Runx2/Wnt/β-catenin	Cardiac hypertrophy
Li et al. [130]	Cardiac stromal cell	miR-21-5p	EV	↑	-	phosphatase and tensin homolog/Akt	HF
Li et al. [131,132,133]	-	lncExACT1	-	↑	-	DCHS2	HF

## 5. Clinical Developments and Applications of Ex-ncRNAs in CVDs

Recently, significant progress has been made in understanding the regulation and roles of Ex-ncRNAs in CVDs and in translating these findings into clinical applications as biomarkers or therapeutic targets [9]. Ex-ncRNAs have been identified as critical regulators in the pathogenesis of CVDs and are thus important candidates for improved diagnosis or prognosis assessment and advanced therapeutics.

### 5.1. Ex-ncRNAs as Biomarkers

Despite the increasing interest in searching for Ex-ncRNA as reliable biomarkers, this field still faces many significant scientific and technical hurdles, including preanalytical and analytical factors that influence data quality and reliability. These factors include sample types, Ex-ncRNA isolation, detection and processing techniques, normalization strategies, and the influence of other cofounders such as drug usage and other CVDs [9,135]. Therefore, the clinical use of Ex-ncRNAs as biomarkers for CVDs is still in its infancy, despite the promising efficacy and feasibility in animal models and a small number of patients as mentioned above. Of note, hsa-Chr8:96, a human homolog of mmu-miR-721, was identified as a novel extracellular small ncRNA for the detection of acute myocarditis in four independent cohorts of patients with myocarditis [136], which demonstrates both the unmet medical need and the enormous promise for developing Ex-ncRNAs as novel biomarkers for CVDs. Currently, more than 10 clinical trials on identifying Ex-ncRNA as novel CVD biomarkers have been initiated or are ongoing, such as ischemic heart diseases (NCT02691286, NCT01875484), atherosclerosis (NCT03279770, NCT03855891), hypertension (NCT04193046), and HF (NCT03345446). A better understanding of the biology of Ex-ncRNAs and the advances in methods for Ex-ncRNA isolation, detection, and analysis, will undoubtedly pave the way for the translation of Ex-ncRNA biomarker research to clinical routine.

### 5.2. Ex-ncRNAs as Therapeutics Targets

Therapeutic targeting of Ex-ncRNAs, including small ncRNAs, lncRNAs, and circRNAs either inside or outside the cells, represents an attractive strategy for treating CVDs. The RNA-targeting approaches include ASOs, siRNAs, shRNAs, antisense, miRNA sponges, CRISPR/Cas9-based DNA editing tools, and CRISPR/Cas13-based RNA editing tools. Notably, siRNAs and ASO have been approved by the United States of America Food and Drug Administration (FDA) and/or the European Medicines Agency (EMA) for clinical use. siRNA is a single- or double-stranded RNA oligo, which exploits the endogenous miRNA pathway to silence mRNAs by loading them into RNA-induced silencing complexes (RISC) for degradation [137]. The successful clinical use of siRNAs targeting proprotein convertase subtilisin/kexin type 9 (PCSK9) mRNAs for lowering circulating LDL cholesterol and then decreasing the CVD risks in human patients has proved the tremendous promise of using siRNAs targeting CVD-causing Ex-ncRNAs, in particular lncRNAs. ASO is a single-stranded DNA oligo with entire or partial complementarity to the target RNAs and may act either by inducing the degradation of target RNAs or by sequestering the interaction of target RNAs with their partner compounds [138]. ASO was first approved by FDA for silencing mRNA in 1998 and has now been widely used to target other ncRNAs, including small ncRNAs [139], lncRNAs [140], and circRNAs [78]. Over the past decades, extensive efforts have been made to clinically utilize siRNAs or ASO to target mRNAs, such as ApoA, PCSK9, Angiotensinogen, FOXO3, and SERCA2A, and only recently target ncRNAs for treating CVDs (Table 3) [141]. CDR132L, a synthetic ASO blocking the functions of miR-132, has entered the Phase II clinical trial for treating patients with reduced left ventricular ejection fraction after MI (NCT05350969). Other strategies, such as antisense, miRNA sponges, and CRISPR/Cas9-based gene editing tools, have also demonstrated promising preclinical effects and are being translated into the clinical routine.

There are also many challenges to be overcome to further the clinical application of RNA-based therapeutics, mainly the specificity and delivery. Virtually all RNA-targeting approaches exploiting the exquisite sensitivity of base-pair complementarity have off-target effects [142]. The quality of RNA therapy is determined by both the efficacy of its on-target effects as well as the minimized off-target side effects, namely specificity. These can be achieved by the development of algorithms for improved RNAi therapeutic design and by chemical modifications. In the past 60 years, hundreds of nucleic acid chemical modifications have been characterized and synthesized, and a number of them have been successfully utilized in RNA therapeutics improving the potency and specificity [143], including the first generation of 2′-deoxy-2′-fluoro (2′-F), 2′-O-methylation (2′-OMe), and the phosphorothioates (PS) backbones, the second generation of 2′-O-(2-methoxyethylation) (2-O-MOE), and the third generation of LNA modification. Efficient and specific delivery of RNA therapeutics to organs/cells still remain the top two greatest challenges in this field [143], owing to their instability, negative charge, hydrophilic nature which prevents diffusion through cell membranes, and rapid cleaning by the liver after systematic administration. The first and second generations of chemical modifications greatly improve the stability and enhance cellular uptake [144]. Specific delivery to targeted cells/tissues can be achieved using modified lipid- and polymer-based nanoparticles, conjugation with different homing ligands, or engineering into specific EVs, among which the endosomal escape of the RNA therapeutics, also termed functional delivery, should be considered. A vast number of other strategies, such as viral vectors with cell type-specific promoters or different envelop proteins, can also be employed for specific and efficient delivery [145].

## 6. Conclusions and Perspectives

**Figure 2:**
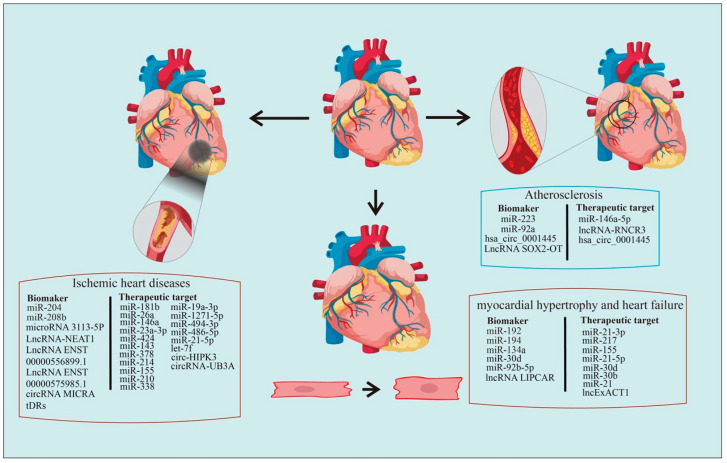
**Extracellular non-coding RNAs as biomarkers or therapeutic targets for cardiovascular diseases.** The summary of Ex-ncRNAs being identified as potential biomarkers or therapeutical targets for cardiovascular diseases.

The poor recovery of cardiac function following heart tissue injury and the lack of effective strategies facilitating this recovery in clinical settings emphasize the importance of preventative monitoring and diagnosis [146]. Ex-ncRNAs, selectively released by different cells, play versatile roles in numerous biological processes and diseases [147]. Recently, studies elucidating the Ex-ncRNAs-mediated intercellular crosstalk in the pathogenesis of various CVDs are emerging [148], and Ex-ncRNAs are increasingly recognized as promising diagnostic biomarkers and druggable targets [149,150]. In this review, we have systematically summarized the regulation and roles of Ex-ncRNAs, including small ncRNAs, circRNAs, and lncRNAs, involved in major cardiovascular events, such as atherosclerosis, ischemic heart diseases, hypertension, cardiac hypertrophy, and HF (Figure 2). We also discussed the strategies and challenges for utilizing Ex-ncRNAs as biomarkers or therapeutical targets.

Although increasing research interests have been attracted to the roles of Ex-ncRNAs during the onset, progression, and prognosis of CVDs [151,152], the utilization of Ex-ncRNAs as biomarkers and therapeutics is still in its infancy in its clinical routine. Due to the heterogeneity of Ex-ncRNAs in different biofluids or even in plasma samples from the same patient at different time points, a robust, reliable, and reproducible readout of specific Ex-ncRNA is indispensable for developing it as a clinical CVD biomarker, which may be achieved by a better understanding of the biology of Ex-ncRNAs and the advances in methods for Ex-ncRNA isolation, detection, and analysis. Similarly, although the specificity of RNA targeting is significantly improved, the specific delivery of RNA therapeutics to desired organs/cells still remains the leading challenge for furthering RNA therapeutics, among which EV-mediated delivery of RNA therapeutics holds the greatest potential. Further understanding of Ex-ncRNA biology and advances in related techniques will eventually establish well-defined diagnostic and therapeutic approaches using Ex-ncRNAs to alleviate the current medical burdens associated with CVDs.

## Figures and Tables

**Table 3:** RNA Therapies Approved or in Clinical Trials for CVDs.

Stage	Drug	Modification and Delivery	Target Gene	Disease	Comments
Clinical trials	AZD8601	naked mRNA	VEGF-A	HF	Phase 2
	CDR132L	ASO	miR-321	HF	Phase 2
	IONIS-AGT-LRx	Antisense	Angiotensinogen	HF	Phase 2
	Olezarsen	Antisense	ApoC-III	Lipid disorders	Phase 3
	Olpasiran	siRNA	Lp(a)	Lipid disorders	Phase 2
	Pelacarsen	Antisense	Lp(a)	Lipid disorders	Phase 3
	Vupanorsen	Antisense	ANGPTL3	Lipid disorders	Phase 2
FDA/EMA approved	Inclisiran	siRNA	PCSK9	Lipid disorders	FDA approved
	Volanesorsen	ASO	APO-C3	Triglycerides	EMA approved

## Data Availability

Not applicable.
